# Supplementary material for: Assessment of hepatic function, perfusion and parenchyma attenuation with indocyanine green, ultrasound and computed tomography in a healthy rat model: Preliminary determination of baseline parameters in a healthy liver
Source: PLoS One. 2021 Dec 17;16(12):e0261447. doi: 10.1371/journal.pone.0261447 (PMC8682902; doi:10.1371/journal.pone.0261447)
Supplement: Attachment — Submitted filename: Response to reviewer_R2.docx [file pone.0261447.s005.docx]

**Academic Editor** Mehmet A Orman

Murcia, Oct 15^th^, 2021

*Revisions of manuscript Ms# PONE-D-20-37951-R1:*

 "Assessment of hepatic function, perfusion and parenchyma attenuation with indocyanine green, ultrasound and computed tomography in a rat model: preliminary standardization of baseline parameters in a healthy liver“.

Dear ladies and gentlemen of the editorial board of PLOS ONE,

We would like to thank the editorial board and the reviewers for their interest shown in our submitted manuscript and their time and effort to review our study. Their valuable comments and critique are greatly appreciated. First of all we wanted to apologize for the delay in the shipment but due to personal problems we have not been able to answer before. We have modified the paper according to the suggestions made; all changes in the text are outlined with red background color. Furthermore, our point-by-point responses to each comment of the reviewers are attached below. The first modified is the title of the manuscript, **“Assessment of hepatic function, perfusion and parenchyma attenuation with indocyanine green, ultrasound and computed tomography in a healthy male rat model: preliminary determination of baseline parameters in a healthy liver”.** Standard English has been revised and edited again following the recommendations of the reviewers.

We hope that all questions were answered to the satisfaction of the Reviewers and the necessary changes implemented. Should any questions remain or further changes to the manuscript deemed necessary, please do not hesitate to contact us.

***Academic editor comments***

**Unfortunately, the 2nd reviewer was not convinced with your response. His main concerns are about the sample size and the validation of the proposed claims with a disease model.**

*As reviewer points out, a low sample size was used in our study but there is a sizeable literature on the reduced sample size use in biomedical experiments, several researchers consider six animals per group as adequate sample size (Kramer M, Font E. Reducing sam-ple size in experiments with animals: historical controls and related strategies. Biol Rev Camb Philos Soc. 2017 Feb;92(1):431-445. doi: 10.1111/brv.12237. Epub 2015 Nov 13. PMID: 26565143; Lenth, 2001; Dell, Holleran & Ramakrishnan, 2002; Devane, Begley & Clarke, 2004; Lewis, 2006; McCrum-Gardner, 2010; Porras N, 2002). Experimental models can indeed be concretized and performed on small batches of animals if everything is very clearly standardized. In our case, the study is performed to provide a rough idea of the standard deviation and the baseline parameters of biochemical, ultrasound and tomographic values of liver function in healthy males Sprague Dawley rats.*

*We think this study might be useful for future studies in pathological models that suffer alterations in the determinations valued as direct and indirect indicators of various situations that involve alterations in liver tissue. The procedures we outline can make a substantial contribution towards the goal of reducing the number of animals used in biomedical research, because understanding normal values of healthy animals could reduce the number of animals required and maximize the information obtained per experiment.*

*In other hand, its true many researchers collect tissue at multiple timepoints to assess changes at the molecular and cellular level in pathology studies, nevertheless, we aimed to establish reference values in healthy Sprague-Dawley rats using different blood test and diagnostic imaging techniques as previously published by other authors. This kind of analysis may be helpful in future research projects to minimize the number of animal samples used and to guide future researchers in similar studies. Our next steps will be to develop and implement these non-invasive techniques to assess different animal models and link our findings in animal research to the clinical practice.*

*We are aware the technology used is not accessible at most institutions, but in order to improve the clinical translation is essential similar technology as clinical studies in animal model. On top, the translational value of animal models could be further enhanced when combined with emerging alternative translational approaches.*

*Checking the baseline values of the present study respect to a pathological model would be of great value and would confirm the data obtained as normalized for subsequent studies. This objective has already been raised by our research group. The next step will be comparing healthy vs different pathological models as future research, like animals with NAFLD, NASH and liver cirrhosis, performing blood parameters, pathological findings and different imaging techniques (US and CT).*

*We support the reviewer´s statement and we will perform future studies focused on find differences between healthy and pathological models. We consider our current manuscript as a pilot study and we aim to keep working on the same direction. The use of multiple non-invasive techniques, including imaging and blood tests, may be highly interesting to find potential differences that may help us to detect and characterize the hepatic parenchyma. This approach would help us finding potential correlations between techniques that in combination have the potential to enhance detection and prediction of liver disease.*

*Even if it is on a very small batch of experienced animals it comes with clear data to follow. Thus, it can be an article that will be read by researchers who want to put evaluations into practice on the same subject, a condition that leads this article to have its place in the chapter of the experimental guide.*
